# *Arabidopsis* tonoplast intrinsic protein and vacuolar H^+^-adenosinetriphosphatase reflect vacuole dynamics during development of syncytia induced by the beet cyst nematode *Heterodera schachtii*

**DOI:** 10.1007/s00709-018-1303-4

**Published:** 2018-09-05

**Authors:** Łukasz Baranowski, Elżbieta Różańska, Izabela Sańko-Sawczenko, Mateusz Matuszkiewicz, Ewa Znojek, Marcin Filipecki, Florian M. W. Grundler, Mirosław Sobczak

**Affiliations:** 1 Department of Botany, Warsaw University of Life Sciences-SGGW, Nowoursynowska 159, 02-766 Warsaw, Poland; 2 Department of Plant Genetics, Breeding and Biotechnology, Warsaw University of Life Sciences-SGGW, Nowoursynowska 159, 02-766 Warsaw, Poland; 3 INRES - Molecular Phytomedicine, Rheinische Friedrich-Wilhelms-University of Bonn, Karlrobert-Kreiten-Straße 13, 53115 Bonn, Germany

**Keywords:** Aquaporins, Cyst nematodes, Feeding site, Lytic vacuoles, γ-TIP, Ultrastructure, V-ATPase

## Abstract

Plant parasitic cyst nematodes induce specific hypermetabolic syncytial nurse cell structures in host roots. A characteristic feature of syncytia is the lack of the central vacuole and the formation of numerous small and larger vesicles. We show that these structures are formed de novo via widening of ER cisternae during the entire development of syncytium, whereas in advanced stages of syncytium development, larger vacuoles are also formed via fusion of vesicles/tubules surrounding organelle-free pre-vacuole regions. Immunogold transmission electron microscopy of syncytia localised the vacuolar markers E subunit of vacuolar H^+^-adenosinetriphosphatase (V-ATPase) complex and tonoplast intrinsic protein (γ-TIP1;1) mostly in membranes surrounding syncytial vesicles, thus indicating that these structures are vacuoles and that some of them have a lytic character. To study the function of syncytial vacuoles, changes in expression of *AtVHA-B1*, *AtVHA-B2* and *AtVHA-B3* (coding for isoforms of subunit B of V-ATPase), and *TIP1;1* and *TIP1;2* (coding for γ-TIP proteins) genes were analysed. RT-qPCR revealed significant downregulation of *AtVHA-B2*, *TIP1;1* and *TIP1;2* at the examined stages of syncytium development compared to uninfected roots. Expression of *VHA-B1* and *VHA-B3* decreased at 3 dpi but reached the level of control at 7 dpi. These results were confirmed for *TIP1;1* by monitoring *At-γ-TIP-YFP* reporter construct expression. Infection test conducted on *tip1;1* mutant plants showed formation of larger syncytia and higher numbers of females in comparison to wild-type plants indicating that reduced levels or lack of TIP1;1 protein promote nematode development.

**Electronic supplementary material:**

The online version of this article (10.1007/s00709-018-1303-4) contains supplementary material, which is available to authorized users.

## Introduction

Plant parasitic cyst-forming nematodes are obligate biotrophic parasites of a number of important crops such as potato (*Solanum tuberosum*), soybean (*Glycine max*), wheat (*Triticum* sp.) and beet (*Beta vulgaris*) throughout the world (Atkinson et al. 2003). Worldwide losses resulting from nematode infestations are estimated at 125 billion US$ per year (Chitwood 2003). Upon root invasion, infective second-stage juveniles (J2) of the nematode induce the formation of a hyperplastic and hypermetabolic syncytium in the root vascular cylinder. Syncytium formation starts from a single cell and expands by successive integration of vascular cylinder cells via formation of local cell wall dissolutions (Grundler et al. 1998; Ohtsu et al. 2017). Characteristic features of syncytia are proliferation of cytoplasm and general increase in the number of organelles. Central vacuoles typical for differentiated parenchymatic vascular cylinder cells become replaced by a system of numerous vesicles of different sizes (Jones and Northcote 1972; Golinowski et al. 1996; Sobczak et al. 1997).

The central vacuole is the largest organelle of differentiated plant cells and its formation is one of the most remarkable features of higher plant cells. However, details of this process are still a matter of discussion (Viotti 2014). One line of evidence implicates a pivotal role of the Golgi apparatus and the trans Golgi network (TGN) in the formation of the central vacuole in vegetative plant cell (Marty 1978, 1999; Robinson and Hinz 1997; Neuhaus and Paris 2005), whereas a second line suggests an important role of the ER system (Amelunxen and Heinze 1984; Hilling and Amelunxen 1985; Robinson and Hinz 1997; Viotti et al. 2013). A single plant cell may contain storage and/or lytic vacuoles, but it is commonly assumed that all types of vacuoles have the same origin (Marty 1999). The presence of the two vacuolar compartments has been evidenced in vegetative (Marty 1999) as well as in embryo storage tissues such as cotyledon cells (Hoh et al. 1995). Storage vacuoles contain proteins or metabolic products (Hoh et al. 1995; Paris et al. 1996; Hara-Nishimura et al. 1998), whereas lytic vacuoles contain hydrolytic enzymes and are functional analogues of animal lysosomes (Jauh et al. 1999; Frigerio et al. 2008). The vacuole is surrounded by a single elementary membrane (tonoplast) that is biochemically different from any other membrane in the plant cell. Tonoplast pyrophosphatase (V-PP_i_ase) and H^+^-ATPase (V-ATPase) are general molecular markers of all types of vacuoles, but they comprise only about 10% of all tonoplastic proteins. V-PP_i_ase and V-ATPase are proton pumps providing a pH gradient and membrane potential between the cell sap and cytosol (Jauh et al. 1999). The V-ATPase complex (700 kDa) is composed of 14 different proteins and consists of a membrane integral complex responsible for proton translocation from cytosol to vacuole and a peripheral complex responsible for ATP hydrolysis (Sze et al. 2002; Cipriano et al. 2008). Tonoplast intrinsic proteins (TIPs) are other key proteins indispensable for functional vacuoles. They belong to the superfamily of the major intrinsic proteins (MIPs) that are membrane channels responsible for the transport of water and small uncharged molecules across membranes. MIPs are localised in membranes of different organelles, show a high diversity in transport specificity and have specific expression patterns in different cell types. There are 35 MIPs in the *A*. *thaliana* genome clustered in four phylogenetic families. The most abundant group of MIPs consists of 13 genes coding for plasma membrane intrinsic proteins (PIPs), whereas TIPs encompass ten, nodulin26-like intrinsic proteins (NIPs) nine, and small basic intrinsic proteins (SIPs) three genes (Alexandersson et al. 2005). TIPs have been described as molecular markers of storage as well as lytic vacuoles (Höfte et al. 1992; Frigerio et al. 2008). A functional TIP (27 kDa) contains six transmembrane domains, five connecting loops, and NH_2_- and COOH-terminal tails (Daniels et al. 1999). In radish (*Raphanus sativus*) roots, TIPs comprise 30–50% of the total tonoplast proteins (Jauh et al. 1999). In *Arabidopsis*, the TIP family is divided into five subgroups (from TIP1 to TIP5). The tonoplast of lytic vacuoles in vegetative tissues contains γ-TIP isoform encoded by *TIP1;1*, *TIP1;2* and *TIP1;3* genes whereas the tonoplast of storage vacuoles in vegetative tissues contains α-TIP and δ-TIP isoforms encoded by *TIP3* and *TIP2* genes, respectively (Paris et al. 1996; Jauh et al. 1999; Saito et al. 2002; Hunter et al. 2007; Frigerio et al. 2008).

As mentioned before, vesicles of various sizes are formed in nematode-induced syncytia instead of a central vacuole. However, the function of these structures has remained unclear. Therefore, we examined the localisation of V-ATPase as a general tonoplast marker protein and γ-TIP as a marker for lytic vacuoles. We also determined the expression patterns of their genes in syncytia induced by the beet cyst nematode *Heterodera schachtii* in *Arabidopsis* roots. Further, we analysed the role of *TIP1;1* in syncytia by an infection assay on a *tip1;1* mutant. Our results provide evidence that small and large vesicles in nematode-induced syncytia are indeed vacuoles and many of them label for the presence of lytic vacuole marker.

## Materials and methods

### Plant material and nematode inoculation

Seeds of the wild-type *Arabidopsis thaliana* L. Heyn. ecotype Columbia (Col-0), mutant *tip1;1* (SAIL_717_D10; N879668 in Col-0 background) and transgenic plants Col-0 expressing γ-TIP-YFP fusion protein under control of native *γ-TIP1;1* promoter (*At-γ-TIP-YFP*; Hunter et al. 2007) were surface-sterilised with 70% (*v*/*v*) ethanol for 1 min, then with 5% (*v*/*v*) sodium hypochlorite (Sigma-Aldrich, St. Louis, MO, USA) for 5 min and rinsed three times for 15 min in sterile distilled water. After sterilisation, five seeds were transferred into Petri dishes (*Ø* 9 cm) containing 20 mL of mineral KNOP medium (pH 6.4) supplemented with 2% (*w*/*v*) sucrose, 0.7% (*w*/*v*) agar (Sigma-Aldrich) and Gamborg’s B_5_ vitamins (Sigma-Aldrich) (Sijmons et al. 1991). The plants were grown at 24 °C under controlled conditions (16/8 light/dark cycles, photon flux intensity 220 μE m^−2^ s^−1^).

*H*. *schachtii* Schmidt cysts were harvested from in vitro stock cultures produced aseptically on white mustard (*Sinapis alba* cv. Albatros) roots grown on the modified KNOP medium. Hatching of juveniles was stimulated by incubating cysts in 3 mM ZnCl_2_. Hatched J2s were collected 7 days later, sterilised in 0.05% (*w*/*v*) HgCl_2_ for 2 min and washed five times in distilled H_2_O (Sijmons et al. 1991). Two-week-old Col-0, transgenic *At-γ-TIP-YFP* line or *tip1;1* mutant plants were inoculated with 100 J2s per root system under sterile conditions. Plates with inoculated plants were kept in a growth chamber under above described conditions.

### Microscopy

After inoculation, roots were inspected under a stereo microscope and infection sites were labelled. At 3, 7 and 14 days post infection (dpi), root segments containing syncytia were fixed in 2% (*v*/*v*) glutaraldehyde (Sigma-Aldrich) and 2% (*w*/*v*) paraformaldehyde (Sigma-Aldrich) in 50 mM sodium cacodylate (Sigma-Aldrich) buffer (pH 7.2) for 2 h. Afterwards, they were washed three times for 10 min with 50 mM cacodylic buffer, and embedded in epoxy resin (Golinowski et al. 1996; Sobczak et al. 1997) for structural transmission electron microscopy or in LR-White resin (Dykstra and Reuss 2003) for immunogold transmission electron microscopy.

### Structural microscopy

Ultra-thin (80 nm thick) sections were taken from epoxy resin-embedded samples using a Leica UCT ultramicrotome (Leica Microsystems, Nussloch, Germany) and collected on formvar-coated copper grids. They were stained with uranyl acetate and lead citrate (Golinowski et al. 1996; Sobczak et al. 1997) and examined under an FEI 268D ‘Morgagni’ (FEI Comp., Hillsboro, OR, USA) transmission electron microscope equipped with 10 MPix Olympus-SIS ‘Morada’ digital camera (Olympus-SIS, Münster, Germany). Collected digital microscopic images were processed for similar contrast and brightness with Adobe Photoshop software.

### Immunocytochemical microscopy

Immunolocalisation for transmission electron microscopy was conducted on 90-nm-thick sections taken from non-osmicated samples embedded in LR-White resin (Dykstra and Reuss 2003). The sections were collected on formvar-coated nickel grids and subjected to immunolocalisation procedure as described in detail by Baranowski et al. (2018). Rabbit anti-subunit E of V-ATPase protein antibody (cat. no. AS09 482 that recognises all three isoforms of VHA-E; Agrisera, Vännäs, Sweden) and anti-γ-TIP protein antibody (cat. no. AS08 327 recognising TIP1;1 protein; Agrisera) were used as primary antibodies. They were detected with goat anti-rabbit immunoglobulin G conjugated to 15-nm colloidal gold particles (British Biocell International, Cardiff, UK). In negative controls, primary antibodies were omitted. Sections were examined under an FEI 268D ‘Morgagni’ transmission electron microscope (FEI Comp.) operating at 80 kV and equipped with a digital camera ‘Morada’ (Olympus-SIS).

### Quantitative RT-qPCR

Total RNA was isolated from uninfected root segments and roots containing syncytia without associated nematodes at 3, 7 and 14 dpi using GeneMATRIX Universal RNA Purification Kit (EURx, Gdańsk, Poland) with additional step of on-column DNase I treatment. RNA concentration, purity and integrity were tested spectrophotometrically with NanoDrop 2000 (Thermo Fisher Scientific, Waltham, MA, USA) or after electrophoretic separation in 1% (*w*/*v*) agarose gels in 1× TBE buffer, they were visualised by SimplySafe (EURx) and photographed. After equalisation of RNA concentrations, cDNA was synthesised using High Capacity cDNA Reverse Transcription Kit (Thermo Fisher Scientific). Primers specific for the studied genes (Supplementary Table S1) were designed using OligoCalc-Oligonucleotide Properties Calculator and Integrated DNA Technologies software based on the alignment of cDNA sequences (NCBI, National Center for Biotechnology, http://www.ncbi.nlm.nih.gov/). AT4G36960 gene encoding RRM (RNA recognition motif-containing protein), which demonstrated the most stable expression in RefSeq tool search within Genevestigator database (Hruz et al. 2008), was used as the endogenous reference. Real-time qPCR were performed in 96-well plates using CFX96 Touch™ Real-Time PCR Detection System (Bio-Rad, CA, USA) according to the manufacturer’s instruction. Four microlitres of 1:25 diluted first-strand cDNA was used as a template in qPCR. Apart from cDNA, each reaction contained 7.5 μL of iTaq Universal SYBR Green Supermix (Bio-Rad), 0.3 μL of each primer (final concentration 0.2 μM) and 2.9 μL of sterile water. Reaction conditions are shown in Supplementary Table S2. Expression of each gene was tested in two biological replicates and three technical repetitions. The specificity of amplified PCR products was verified by melting curve analysis. For statistical analysis, the calculation of reaction efficiency was performed using LinRegPCR software (Ramakers et al. 2003) whereas the absolute normalised gene expression levels and statistical significance of their differences were calculated using REST2009 software (Pfaffl et al. 2002).

### Expression of *TIP1;1* gene in *At-γ-TIP-YFP* line

The seeds of transgenic *A*. *thaliana* plants expressing *At-γ-TIP-YFP* reporter construct (Hunter et al. 2007) were grown on KNOP medium in Petri dishes (*Ø* 6 cm) and inoculated as described above. Nematode-infected (3, 7 and 14 dpi) and uninfected roots of *At-γ-TIP-YFP* plants were in vivo examined using a Leica TCS SP5 II (Leica Microsystems) confocal laser scanning microscope (*λ*_exc_ = 514 nm; *λ*_em_ = 520–525 nm).

### Nematode infection test

Col-0 and *tip1;1* plants were grown and inoculated as described above. The numbers of males and females per plant, and sizes of syncytia and associated female nematodes were counted and measured at 14 dpi. The experiments were repeated three times with ten plants per genotype. For both lines, 50 syncytia associated with females were randomly selected and photographed using a Leica M165C (Leica Microsystems) stereo microscope equipped with a Leica DFC 425 (Leica Microsystems) digital camera. The syncytia and females were outlined and their areas were measured using a Leica Application Suite software (V3.8) (Leica Microsystems). Data were analysed using Student’s *t* test (*p* < 0.05).

## Results

### Development of vacuoles in syncytium

**Fig. 1:**
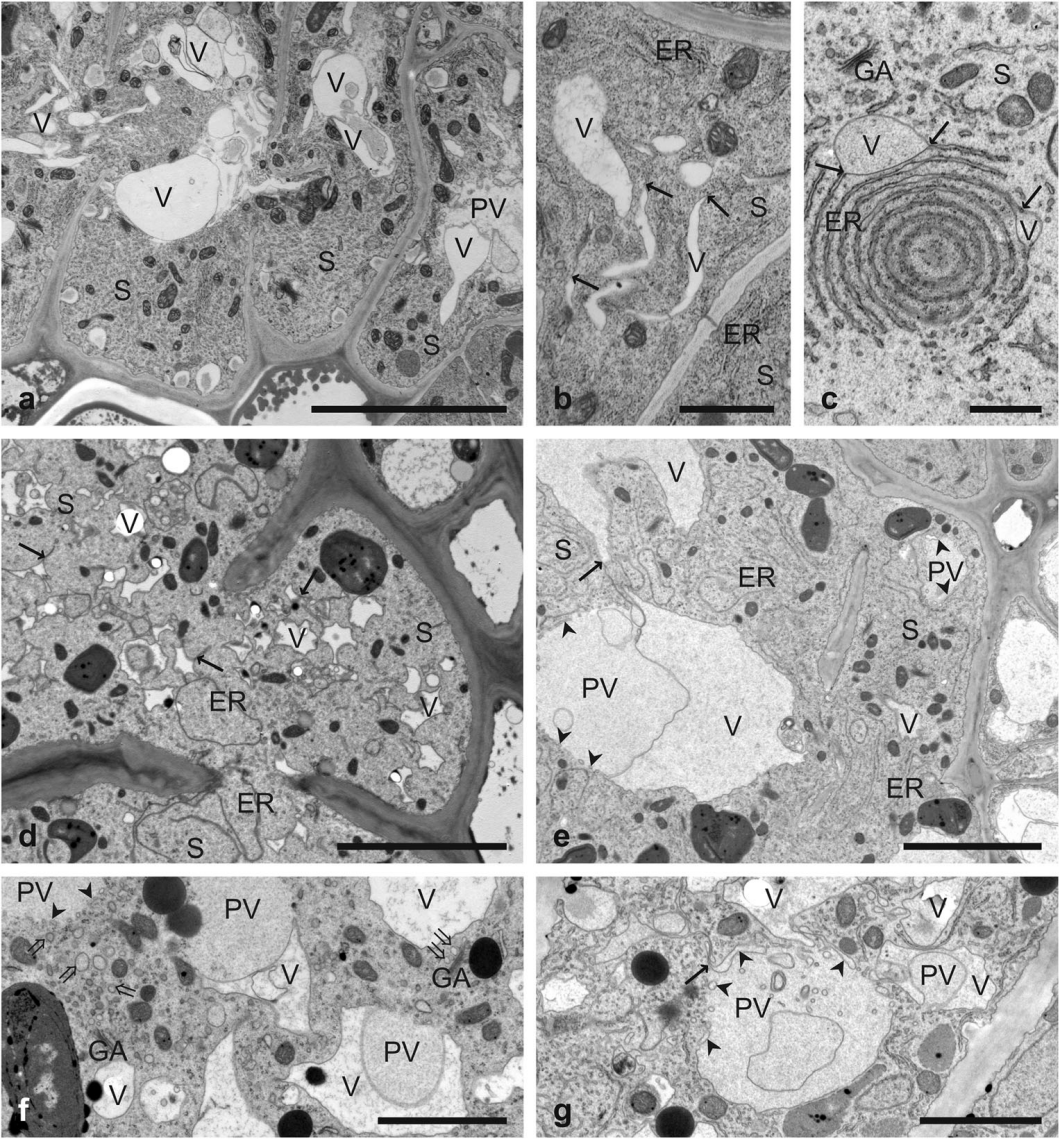
Development of vacuoles in nematode-induced syncytia. Transmission electron microscopy micrographs of cross sections of syncytia collected at 3 (**a**, **b**), 7 (**f**, **g**) and 14 (**c**–**e**) dpi. **a** Re-differentiation of the central vacuole in recently incorporated syncytial elements. **b**, **c** New small cytoplasmic vacuoles are formed via dilation of ER cisternae. **d** Accumulation of small vacuoles in syncytial element. **e**–**g** Formation of organelle-free pre-vacuole regions and large vacuoles. Arrows point to junctions of tonoplast and ER cisternae, arrowheads point to tubules and flattened cisternae at the interface between electron translucent organelle-free pre-vacuole region and cytoplasm, and double tail arrows indicate Golgi apparatus-derived vesicles. *ER*, cisternae of endoplasmic reticulum; *PV*, electron translucent organelle-free pre-vacuole region; *S*, syncytium; *V*, vacuole. *Bars* 1 μm (**b**, **c**), 2 μm (**f**, **g**) and 5 μm (**a**, **d**, **e**)

One of the most remarkable ultrastructural features of nematode-induced syncytium is the absence of the central vacuole. The vacuole is re-differentiated in cells incorporated into syncytium prior or shortly after their integration into a syncytium (Fig. 1a). The details of this process have never been described yet. Accumulation of numerous vacuoles/vesicles surrounded by a single membrane or electron translucent post-vacuolar regions were frequently found in central regions of newly incorporated syncytial elements located in distal/terminal regions of syncytia. Unfortunately, it was impossible to follow the process of central vacuole re-differentiation in sufficient detail to provide a precise description. Concomitantly with re-differentiation of the central vacuole, new small vacuoles/vesicles were formed de novo in the paramural regions of syncytial cytoplasm in elements located in the deeper regions of syncytia and close to nematode’s head (Fig. 1b, c). They were formed apparently by the dilation of the ER cisternae and swelling of their lumens. These vacuoles/vesicles were usually irregularly shaped and they were formed both, in young (Fig. 1b) and old syncytia (Fig. 1c, d). They appeared abundantly only in some syncytial elements whereas other elements remained almost free of them (Fig. 1d). After the first moult of the developing nematode from J2 to J3 at ca. 6 dpi, also larger vacuoles were formed in the syncytia. Their development was preceded by the formation of electron translucent pre-vacuole regions that were free of organelles (Fig. 1e–g) and resembled a so-called ‘zone of exclusion’ differentiating during regular development of the central vacuole (Amelunxen and Heinze 1984; Hilling and Amelunxen 1985; Robinson and Hinz 1997). At their interface with the cytoplasm, small tubular or flattened cisternae accumulated (Fig. 1e–g). They appear to be cross-sectioned dilated tubules and cisternae of the ER as their connections with regular ER structures can be seen (Fig. 1e, g). However, also dictyosomes and their secretory vesicles were often found next to the electron translucent pre-vacuole regions (Fig. 1f), suggesting that they could accumulate at the border of the pre-vacuole regions and contribute directly, or indirectly via TGN, to the development of larger vacuoles in syncytia. Neighbouring tubules and vesicles fused together pushing the flocky material present in the electron translucent pre-vacuole regions back to the cytoplasm and a vacuole lumen was formed inside fused vesicles (Fig. 1e, f). No recognisable ultrastructural and anatomical differences between syncytia induced in wild-type Col-0 plants and *tip1;1* mutant were observed (Supplementary Figure S1).

### Immunolocalisation of V-ATPase subunit E and γ-TIP1;1 proteins

**Fig. 2:**
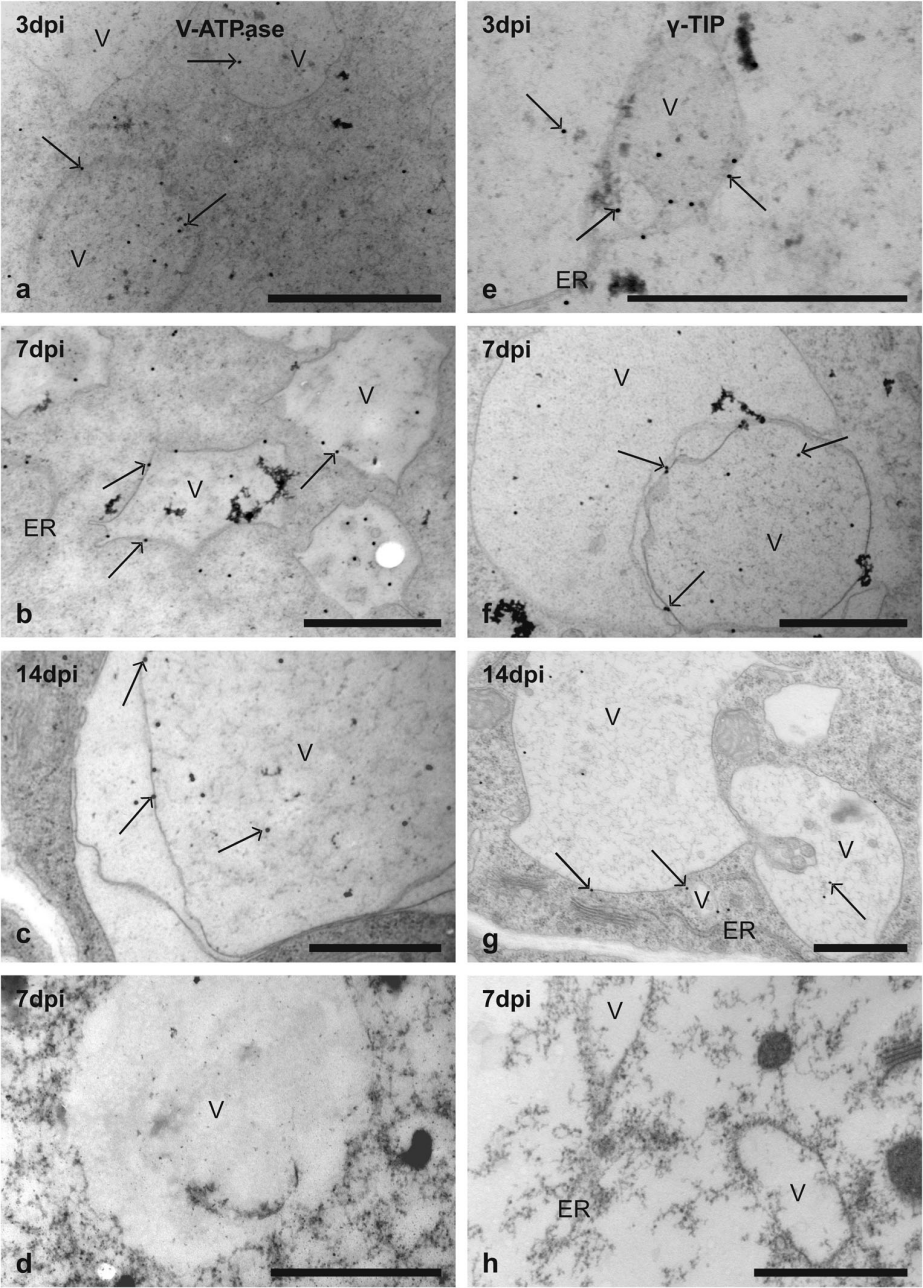
Immunogold transmission electron microscopy localisation of γ-TIP and V-ATPase proteins in syncytia induced in wild-type plants (Col-0). Images of cross sections of *Arabidopsis* root containing syncytia at 3 (**a**, **e**), 7 (**b**, **d**, **f**, **h**) and 14 (**c**, **g**) dpi incubated with anti-V-ATPase subunit E (**a**–**c**) and anti-γ-TIP1;1 (**e**–**g**) antibodies. Images of control sections from labelling experiments where primary antibodies were omitted (**d**, **h**). Arrows indicate randomly selected gold grains. *ER*, endoplasmic reticulum; *V*, vacuole. *Bars* 1 μm (**a**–**h**)

The E subunits of V-ATPase complex that is a general marker for tonoplast membrane and γ-TIP protein that is a generally accepted marker for the tonoplast of lytic vacuoles were both immunolocalised on ultra-thin cross sections of uninfected roots and roots containing 3-, 7- and 14-dpi syncytia. Immunogold labelling showed the presence of V-ATPase subunit E protein in the vascular cylinder, cortex and rhizodermis cells of uninfected roots. In syncytia at 3, 7 and 14 dpi, gold grains indicating V-ATPase were found in small (Fig. 2a, b) and large vacuoles (Fig. 2c). However, the gold grains were found mainly in the lumen of the vacuole and only a few were localised to the tonoplast (Fig. 2a–c). Few gold grains indicating the presence of V-ATPase subunit E protein were found also in the syncytial cytoplasm (Fig. 2a–c).

In uninfected *Arabidopsis* roots, the colloidal gold grains indicating the presence of γ-TIP1;1 protein were found only in cells differentiating into xylem vessels. At all examined time points of syncytium development, gold grains localised γ-TIP protein predominantly in membranes surrounding small (Fig. 2e, g) and larger vacuoles (Fig. 2f, g) and to a lesser extent in their lumen (Fig. 2e–g). Gold grains were also found attached to the membranes of ER and in syncytial cytoplasm (Fig. 2e, g). In control labellings, without primary γ-TIP1;1 or V-ATPase subunit E antibodies, no gold grains were found in any syncytial compartment (Fig. 2d, h).

### Analysis of *V-ATPase* and *TIP* expression during syncytium development

To get insight into the role of *V-ATPase* and *TIP* genes in syncytium development and maintenance, a bioinformatic meta-analysis was performed with transcriptomic microarray and RNA-seq data (Szakasits et al. 2009; Matuszkiewicz et al. 2018; Supplementary Table S3). Based on these data, almost all of differentially expressed *TIPs* were downregulated (*γ-TIP1;1*, *γ-TIP1;2*, *δ-TIP2;1*, *δ-TIP2;2*, *δ-TIP2;3* and *ε-TIP4;1*) in syncytia. Only *β-TIP* (*TIP3;2*) was upregulated, but its absolute expression was on a very low level. Most genes coding for V0 and V1 subunits of V-ATPase were stably expressed in syncytia except of *VHA-B1* and *VHA-B3* that were found to be downregulated in RNA-seq data (Matuszkiewicz et al. 2018; Supplementary Table S3).

**Fig. 3:**
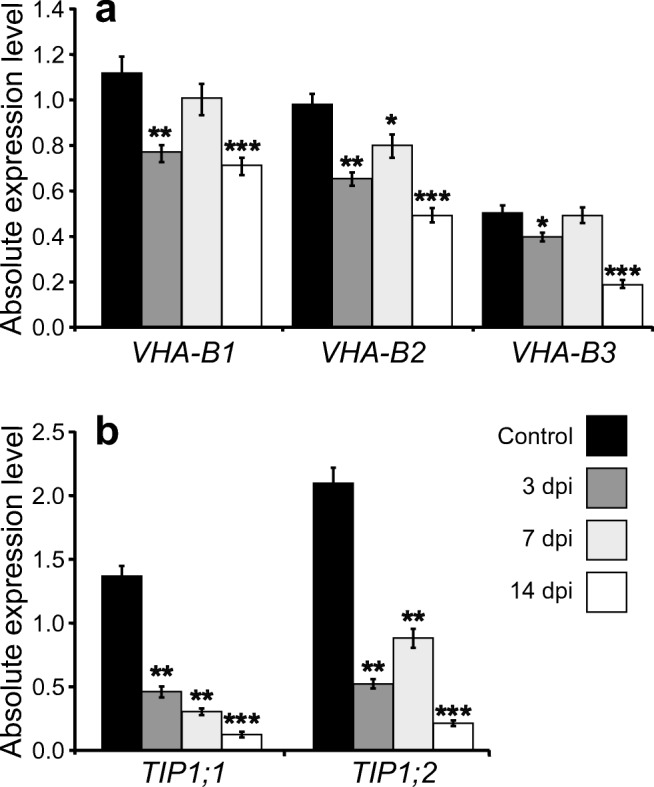
Analyses of expression levels of V-ATPase subunit B (*VHA-B*) and *γ-TIP* genes in wild-type Col-0 *Arabidopsis* roots. Absolute normalised expression levels of *VHA-B* (*VHA-B1*, *VHA-B2* and *VHA-B3*) genes (**a**) and *γ-TIP* (*TIP1;1* and *TIP1;2*) genes (**b**) transcripts in syncytia at 3, 7 and 14 dpi in comparison to uninfected roots. Statistical analysis was performed using LinRegPCR (calculation of reaction efficiency) (Ramakers et al. 2003) and REST2009 (calculation of absolute gene expression level and statistical significance of their differences) (Pfaffl et al. 2002). Bars represent mean values ±SE (*n* = 6). Asterisks above the bars indicate statistically significant differences in comparison to the uninfected roots at *p* < 0.05 (*), *p* < 0.01 (**) or *p* < 0.001 (***)

Using RT-qPCR, we confirmed that *TIP1;1*, *TIP1;2*, *TIP1;3*, *VHA-B1; VHA-B2* and *VHA-B3* genes were generally downregulated at three selected time points corresponding to different stages of syncytium development (3 dpi—early stage of syncytium development, 7 dpi—well-established syncytium, and 14 dpi—mature syncytium) (Fig. 3). *TIP1;3* expression was at the detection limit; thus, it was omitted from further analysis. Genes encoding vacuolar markers from γ-TIP family (*TIP1;1* and *TIP1;2*) and different isoforms of subunit B of the V-ATPase (*VHA-B1*, *VHA-B2* and *VHA-B3*) were found to be significantly downregulated at 3 and 14 dpi (Fig. 3). At 7 dpi, expression of *VHA-B1* and *VHA-B3* was at the level observed in the control, while expression of *VHA-B2* was still lower than that in uninfected roots (Fig. 3a). The strongest downregulation was found for *γ-TIP* genes in syncytia at 14 dpi (Fig. 3b). The transcriptional suppression of *VHA-B1* (− 0.45 log_2_FC) and *VHA-B3* (− 0.67 log_2_FC) at 14 dpi was less pronounced compared to changes in expression of *γ-TIPs* (Supplementary Table S3). The suppression of *TIP1;2*, *VHA-B1* and *VHA-B2* seems to act in two stages with the strongest effect at 3 and 14 dpi (Fig. 3).

### Expression of *γ-TIP1;1* gene in *At-γ-TIP-YFP* line

**Fig. 4:**
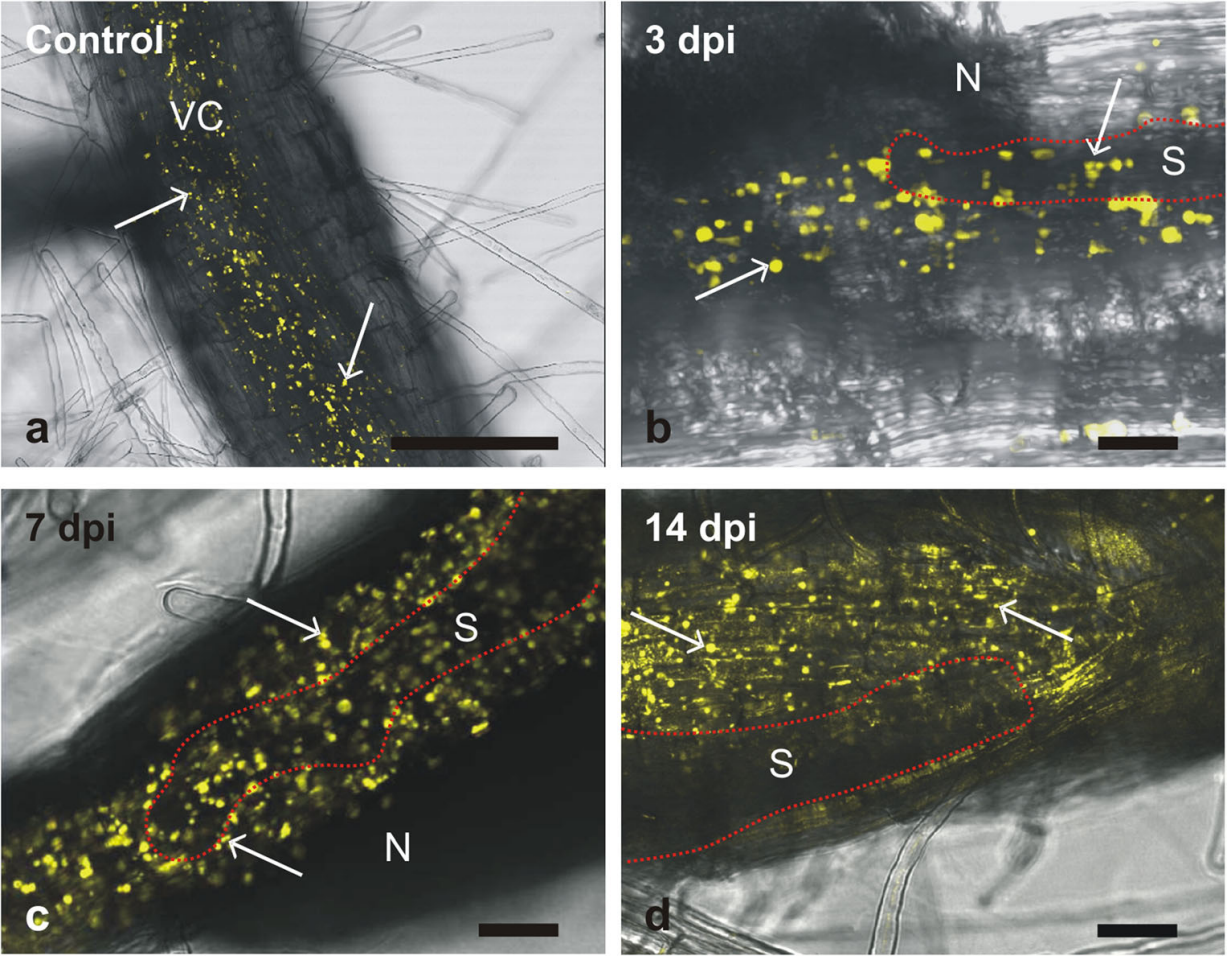
Analyses of *γ-TIP1;1* expression in roots of *At-γ-TIP1;1-YFP* transgenic plants. Confocal laser scanning microscopy images of γ-TIP-YFP fusion protein depositions in uninfected roots (**a**) and roots containing syncytia at 3 (**b**), 7 (**c**) and 14 (**d**) dpi. Yellow fluorescence indicates expression of γ-TIP-YFP protein and some selected γ-TIP-YFP depositions are indicated by arrows. The syncytia are outlined by red dotted lines (**b**–**d**). Each image is an overlay of single fluorescent image projection (*λ*_exc_ = 514 nm; *λ*_em_ = 520–525 nm) merged with transmitted light channel. *N*, nematode; *S*, syncytium; *VC*, vascular cylinder. *Bars* 20 μm (**a**–**d**)

To confirm changes in the expression pattern of the most strongly downregulated *γ-TIP1;1* gene, we examined its expression in vivo using confocal laser scanning microscopy and transgenic *Arabidopsis* plants expressing *γ-TIP1;1-YFP* reporter construct under control of the native *γ-TIP1;1* promoter. In uninfected roots, *γ-TIP1;1-YFP* was expressed in older parts of the roots as described by Hunter et al. (2007), but the presence of fusion protein was restricted only to small spherical structures in the vascular cylinder cells (Fig. 4a), whereas the tonoplast of the central vacuoles of other root cells remained unlabelled. This is not in line with the localisation pattern of γ-TIP-YFP fusion protein described by Hunter et al. (2007). In root samples containing syncytia at 3 dpi, the fluorescence signal of γ-TIP-YFP fusion and number of spherical structures containing γ-TIP-YFP was high near to the head of the nematode (Fig. 4b), but in the regions corresponding to the growing syncytium, it showed an apparently less intense signal as illustrated by weaker fluorescence and smaller sizes of labelled structures as compared to neighbouring root regions. This pattern was even better visible in the 7- and 14-dpi syncytia (Fig. 4c, d). These results are in a general agreement with our RT-qPCR results (Fig. 3).

### *tip1;1* mutation influences nematode parasitism

**Fig. 5:**
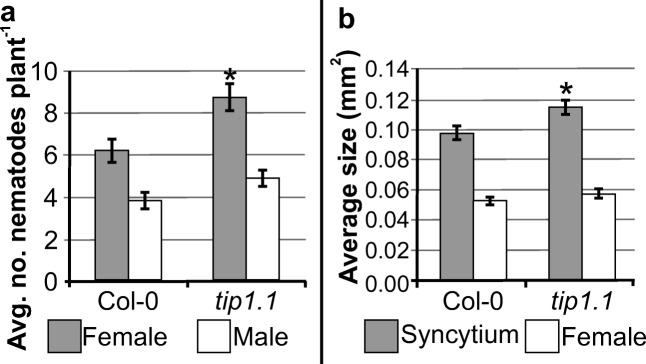
Nematode infection assay. Development of *H*. *schachtii* and nematode-induced syncytia in roots of wild-type Col-0 and *tip1;1* mutant plants. **a** Average number of females and males developed at 14 dpi. **b** Average sizes of syncytia and associated females at 14 dpi. Data represent means (±SEM; standard error of mean) from three independent experiments, each containing 10 plants per genotype. Asterisks indicate significant differences from the wild-type plants according to Student’s *t* test (*p* < 0.05)

To link the role of TIP1;1 with the development of feeding sites induced by *H*. *schachtii* in *Arabidopsis* roots, infection and nematode development tests were performed on loss-of-function *tip1;1* mutant line. The average number of females developed on *tip1;1* mutant roots was significantly higher compared to that of wild-type plants (Fig. 5a). The number of developed males was also higher, but this difference was insignificant (Fig. 5a). The average size of syncytia associated with females of the nematode differed significantly between both lines and it was in average about 17% larger in *tip1;1* mutant than in Col-0. The size of females was not significantly affected (Fig. 5b).

## Discussion

The development of nurse cells induced by cyst-forming nematodes starts from a single initial syncytial cell (ISC) selected by the infective juvenile at the end of its migration (Wyss 1992; Golinowski et al. 1996). Thereafter, cells next to the ISC are modified and fused with the ISC via formation of partial cell wall dissolutions (Grundler et al. 1998; Ohtsu et al. 2017) thus forming a syncytium with a confluent protoplast. One of the most intriguing questions at this stage is what the decisive host’s cell features are that a particular cell becomes selected as the ISC or it becomes incorporated into the nematode-induced syncytium. One of the aspects to be considered at the cytological and cytopathological level is whether the ISC and cells incorporated into syncytia contain a fully differentiated central vacuole. A few images published up today suggest that the ISC does contain the regular central vacuole (Grundler et al. 1997, 1998; Sobczak and Golinowski 2009) that is soon re-differentiated and replaced by numerous small ones (Magnusson and Golinowski 1991; Golinowski et al. 1996; Sobczak and Golinowski 2009). In 3-day-old syncytia, the central vacuoles are generally missing and only their remnants and small vacuoles are present. Unfortunately, we were unable to provide a detailed and univocal description of how the central vacuole is re-differentiated and whether this process occurs before incorporation of the ‘candidate’ cell into syncytium or after its incorporation. Here, we show apparently fragmented remnants of central vacuoles in recently incorporated apical syncytial elements (Fig. 1a). It therefore seems that the central vacuole is re-differentiated after the cell is incorporated into the syncytium. However, this process is rather rapid as it could be observed in a very limited number of samples and additionally no single set of ultrastructural changes could be consistently observed. The vacuolar system in young syncytia seems to be composed of few small vacuoles being remnants of the central vacuole, while numerous small vacuoles are formed by local widening of the ER cisternae. This observation does not fit to generally accepted models of vacuole differentiation in vegetative plant cells (Marty 1978, 1999; Amelunxen and Heinze 1984; Hilling and Amelunxen 1985; Robinson and Hinz 1997; Neuhaus and Paris 2005; Viotti et al. 2013). However, as suggested by Viotti (2014), cytodifferentiation mechanisms leading to formation of the first central vacuole may not operate any longer in fully differentiated cells. Although a nematode-induced syncytium is formed from apparently differentiated vascular cylinder cells, it reveals many ultrastructural features typical for meristematic cells, such as electron dense cytoplasm, lack of central vacuoles, plastids resembling proplastids, enlarged nuclei undergoing endoreduplication of their DNA, as well as numerous ribosomes and ER structures (Jones and Northcote 1972; Golinowski et al. 1996; Sobczak and Golinowski 2009). Thus, we hypothesise that during early stages of syncytium development (until ca. 7 dpi), small vacuoles are formed via direct swelling of the ER cisternae as the simplest and most basic mechanism of vacuole formation. At more advanced stages of syncytium development, when the syncytium matures (after ca. 7 dpi), the syncytial vacuoles are formed via more generally accepted pathway including formation of ‘zones of exclusion’ and organelle-free pre-vacuole regions (Amelunxen and Heinze 1984; Hilling and Amelunxen 1985; Robinson and Hinz 1997; Viotti et al. 2013; Viotti 2014). Unfortunately, although extensive efforts have been undertaken, we were unable to precisely identify the TGN network (Marty 1978, 1999) and analyse its potential contribution to the development of syncytial vacuoles.

In spite of commonly accepted claim that syncytium contains numerous small vacuoles replacing the central vacuole typical for differentiated plant cells, no other evidence except for morphological similarity to vacuoles at transmission electron microscopy images have been available. In typical parenchymatic plant cell, the central vacuole is the largest organelle that plays important roles in storage of proteins and metabolic products, lytic processes, programmed cell death (PCD), regulation of cell volume and turgor pressure, maintenance of pH level, regulation of ion concentrations in the cytosol, signal transduction and many others (Paris et al. 1996; Jauh et al. 1999; Andreev 2001). These functions largely overlap with processes being re-programmed in developing syncytia, what is known from transcriptomic studies (Szakasits et al. 2009; Cabrera et al. 2014; Matuszkiewicz et al. 2018). Tonoplast (a single membrane separating cell sap from the cytosol) contains specific marker proteins such as V-ATPase and TIPs, which regulate and facilitate its functions. V-ATPase is a general marker for tonoplast membrane (Kluge et al. 2004). TIPs are also molecular markers of the tonoplast, but different TIPs are markers of different vacuolar compartments. Two separate vacuolar compartments, storage and lytic, may operate in the same vacuole at the same time (Paris et al. 1996; Jauh et al. 1999; Frigerio et al. 2008). Tonoplast of storage vacuoles in vegetative cells contains α-TIPs and δ-TIPs, whereas γ-TIPs are present in tonoplast of lytic vacuoles (Hoh et al. 1995; Marty-Mazars et al. 1995; Frigerio et al. 2008). Using immunogold cytochemistry and antibody recognising all VHA-E isoforms that may serve similarly to B subunit of V-ATPase as a convenient indicator of V-ATPase abundancy because they both belong to the same V1 peripheral complex of V-ATPase (Cipriano et al. 2008), we have shown that syncytial vesicles are indeed vacuoles. Similarly, immunolabelling with anti-γ-TIP1;1 antibody showed the lytic nature of many small syncytial vacuoles. However, a clear relationship between the type of vacuole and type of TIP proteins present in its tonoplast was denied (Hunter et al. 2007; Olbrich et al. 2008). It was shown that α-TIPs, δ-TIPs and γ-TIPs are present in the same tonoplast indicating that the presence of a specific form of TIP protein is not discriminative for assessing function of a particular vacuole.

Expression analyses of *AtVHA-B* genes showed their general downregulation during development of syncytia. Similarly, the expression level of *γ-TIPs* genes coding for putative markers of lytic vacuoles decreased during syncytium development. The changes in expression of *TIP1;1* gene were also confirmed by in vivo analyses of *γ-TIP1;1-YFP* construct expressing fluorescently labelled TIP1;1 protein under control of its native promoter in transgenic *Arabidopsis* plants. Expression and accumulation of TIP1;1 in syncytia during their development was reduced and they contained fewer and smaller spherical structures accumulating γ-TIP-YFP fusion protein that resembled those described in root cells (Hunter et al. 2007) and in hypocotyl and cotyledon epidermis of young *Arabidopsis* seedlings (Saito et al. 2002). However, in our observations, we did not detect fusion protein localised to the tonoplasts of the central vacuoles. Lower expression and abundancy of vacuolar markers agree with the general view that the volume of syncytial vacuole decreases during syncytium development (Jones and Northcote 1972; Magnusson and Golinowski 1991; Golinowski et al. 1996). However, the role of TIPs in plant-nematode interactions has never been examined in detail with the exception of research of Xue and co-workers (Xue et al. 2013) showing that Mi8D05 effector of root knot nematode, *Meloidogyne incognita*, interacts with tomato TIP2;3 protein to facilitate giant cell development via control of water transport. It was also shown that *Fusarium oxysporum* infection decreased expression of 19 membrane intrinsic proteins (MIPs) in chickpea (*Cicer arietinum* L.) to regulate water balance and to promote fungus infection (Deokar and Tar’an 2016) and that AtTIP1 and AtTIP2 proteins influenced replication and development of cucumber mosaic virus (CaMV) by interaction with viral protein (Kim et al. 2006).

Decreased expression of studied genes may reflect not only smaller size of syncytial vacuoles, but also physiological changes in syncytium that is extensively explored by the nematode as source of food. Since TIP proteins are involved in water transport, the observed differences concerning the numbers of developed females and larger sizes of their syncytia in *tip1;1* mutant in comparison to control plants may be related to their turgor pressure that is an important parameter responsible for proper syncytium functioning (Böckenhoff and Grundler 1994). The mechanism how exactly downregulation of aquaporins might increase the turgor pressure may be related to the complex biology of syncytia. Considering that aquaporins allow water transport in both directions (import/export), the final effect of their action depends on other factors regulating water flux such as osmolytes and their transporters (Argiolas et al. 2016). It is also possible that the net effect of aquaporins depends on their activity balance in several subcellular locations. It is worthy also to recall high turgor pressure in syncytia and their symplasmic isolation from surrounding tissues (Böckenhoff and Grundler 1994). Relying on the transcriptomic data (Supplementary Table S3; Szakasits et al. 2009; Matuszkiewicz et al. 2018), we found extensive downregulation of aquaporins in wild-type syncytia including those localised to tonoplast (TIPs) as well as plasma membrane (PIPs; Supplementary Table S3). This suggests the existence of a conserved mechanism regulating transcription of all aquaporins genes. We hypothesise that overall downregulation of *PIPs* and *TIPs* restricts the water loss making a nematode the main water and metabolites sink. At the same time, water uptake may be facilitated by upregulation of sugar transporters causing increased import of osmotically active sugars into syncytium from phloem (Hofmann et al. 2007, 2009) and increased plasma membrane area on syncytium cell wall regions adjacent to the vessels where cell wall ingrowths are abundantly formed (Jones and Northcote 1972; Golinowski et al. 1996). It seems that syncytia retain more sugars and other osmolytes in the cytosol, which is a food source for developing nematode, than in the cell sap. The downregulation of *SWEET16* and *SWEET17*, coding for vacuolar sugar transporters, supports such assumption (Hedrich et al. 2015; Szakasits et al. 2009). In addition to downregulation of vacuolar aquaporins, sugar importers and V-ATPases, the syncytia show progressive decrease in the transcript level of many proteases including vacuolar VPEG, cysteine proteinase RD21A and γ-glutamyl hydrolase. Together with observed decreasing labelling with *γ-TIP1;1-YFP* fusion protein in developing syncytia and lack of anatomical and ultrastructural differences between syncytia induced in wild-type and *γ-tip1;1* mutant plants (Supplementary Figure S1), it indicates decreasing role of vacuoles and lytic vacuoles in syncytium development in particular. This is also in line with recently reported downregulation of autophagy markers, such as metacaspases and autophagy-related genes (*ATG*), in developing syncytia (Matuszkiewicz et al. 2018) and might indicate anti-autophagic mechanism in syncytia that apparently involves vacuoles. The gradual disappearance of lytic vacuoles does not mean that they do not contribute to syncytium development and functioning at earlier stages. Stronger decrease in *γ-TIP1;1* and *γ-TIP1;2* transcript levels than in the case of *VHA-B1* and *VHA-B2* (*VHA-B3* did not change significantly) may lead to increase of V-ATPase density in syncytial tonoplast leading to stronger acidification of small syncytial vacuoles and allow more efficient digestion of proteins present there at a low pH level. Such modulation of V-ATPase density is a well-known proton pump regulatory mechanism in human renal epithelial cells (Cipriano et al. 2008). We speculate that switching off one of the vacuolar aquaporins in the *tip1;1* mutant may lead to the enhanced release of free amino acids to the cytosol as a result of effective proteolysis in vacuole and limited possibility of balancing increasing amino acid concentration by water intake into small syncytial vacuoles. Among these amino acids, there is certainly l-glutamine that is one of the strongest osmolytes in the cell (Argiolas et al. 2016), thus increasing water potential of syncytium protoplast and consequently turgor of syncytia as evidenced by Böckenhoff and Grundler (1994). Such a mechanism is likely since the upregulations of vacuolar amino acid transporters (e.g. *AVT3A*) and glutamine markers (e.g. *ASN3*, *GLN1*) were shown in transcriptomic analyses of syncytia (Szakasits et al. 2009; Matuszkiewicz et al. 2018). The above discussion on vacuole role in syncytium development and functioning recalls that analyses of vacuole-type markers transcript levels should be interpreted with special caution since the variation in tonoplast area and cell sap volume can substantially influence functioning of vacuoles (Béré et al. 2017).

The expression patterns of *γ-TIP* and *V-ATPase* genes and immunolocalisation of their proteins confirm the vacuolar character of small vesicles present in syncytia while the presence of γ-TIP in their membranes suggests lytic character of at least some syncytial vacuoles. We show that the syncytial vacuole changes dynamically during feeding site development. TIP1;1 turned out to be an important negative regulator of syncytium development and functioning that possibly acts via turgor pressure modulation.

## Electronic supplementary material

ESM 1 (PDF 381 kb)

ESM 2 (PDF 107 kb)

ESM 3 (PDF 94.4 kb)

ESM 4 (PDF 64.8 kb)

## Abbreviations

dpi: Days post inoculation
ER: Endoplasmic reticulum
ISC: Initial syncytial cell
RT-qPCR: Reverse transcription-quantitative polymerase chain reaction
TIP: Tonoplast intrinsic protein
V-ATPase: Vacuolar H^+^-adenosinetriphosphatase (H^+^-ATPase)
