# Supplementary material for: Arabidopsis tonoplast intrinsic protein and vacuolar H+-adenosinetriphosphatase reflect vacuole dynamics during development of syncytia induced by the beet cyst nematode Heterodera schachtii
Source: Protoplasma. 2018 Sep 5;256(2):419–29. doi: 10.1007/s00709-018-1303-4 (PMC6510842; doi:10.1007/s00709-018-1303-4)
Supplement: Supplementary file 1 — (PDF 381 kb) [file 709_2018_1303_MOESM1_ESM.pdf]

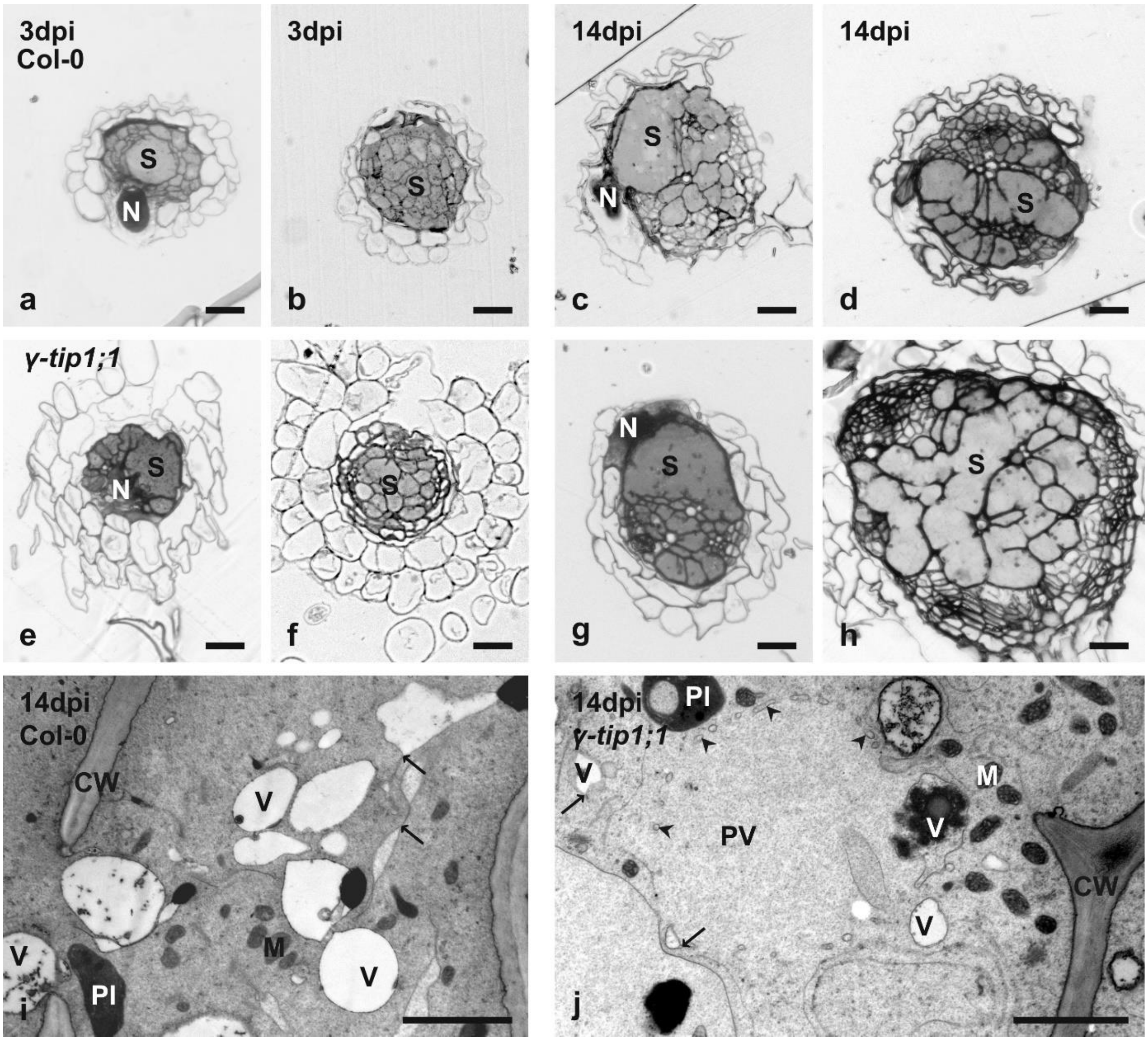

Supplementary Figure S1 Anatomy and ultrastructure of nematode-induced syncytia. Light (**a**-**h**) and transmission electron microscopy (**i**, **j**) micrographs of cross sections of syncytia induced in wild-type Col-0 ecotype (**a**-**d**, **i**) and *tip1;1* mutant (**e**-**h**, **j**) roots and collected at 3 (**a**, **b**, **e**, **f**) and 14 (**c**, **d**, **g**-**j**) dpi. The sections were taken close to the nematode's head (**a**, **c**, **e**, **g**) or in the middle part of syncytia (**b**, **d**, **f**, **h**-**j**). Arrows point to junctions of tonoplast and ER cisternae, arrowheads point to tubules and flattened cisternae at the interface between electron translucent organelle-free pre-vacuole region and cytoplasm. *CW* cell wall, *M* mitochondrion, *N* nematode, *Pl* plastid, *PV* electron translucent and organelle-free pre-vacuole region, *S* syncytium, *V* vacuole. *Bars* 20 µm (**a**-**h**), 2 µm (**i**, **j**)
