# Supplementary material for: Arabidopsis tonoplast intrinsic protein and vacuolar H+-adenosinetriphosphatase reflect vacuole dynamics during development of syncytia induced by the beet cyst nematode Heterodera schachtii
Source: Protoplasma. 2018 Sep 5;256(2):419–29. doi: 10.1007/s00709-018-1303-4 (PMC6510842; doi:10.1007/s00709-018-1303-4)
Supplement: Supplementary file 2 — (PDF 107 kb) [file 709_2018_1303_MOESM2_ESM.pdf]

Supplementary Table S1 List of primers used in experiments

Gene ID	Primer	Sequence
VHA-B1 (At1g76030)	VAB1f	CCAGGAGCTGCTTTGATTTGG
	VAB1r	CTGGTACTTTGGACCCTTCACT
VHA-B2 (At4g38510)	VAB2f	CGAATTAAATCTAAAATGGGTGCTG
	VAB2r	CGGGTAGTTCCATCCCCAAG
VHA-B3 (At1g20206)	VAB3f	GCCACACCACCTTCATCAAAC
	VAB3r	CAAACCACTCCACAATCCCTCT
γ-*TIP1;1* (At2g36830)	TIP1;1f	GCCAACATCTCTGGTGGACA
	TIP1;1r	AAAAGCCGGCACAGCCAAG
γ-*TIP1;2* (At3g26520)	TIP1;2f	ACATCACTCTCCTCCGTGGT
	TIP1;2r	GAACGCTGGAATTGGCTCGC
γ-*TIP1;3* (At4g01470)	TIP1;3f	ACATTCTGGTTGGTGGTGCT
	TIP1;3r	TGGCTGCACCGATAAAAGGA
AtRRM (AT4G36960)	AtRRMf	TCGAATCCCTTCATCAGTCTC
	AtRRMr	CTGTTATTTCCCCATACCTCTCA
γ-*TIP1;1* (At2g36830) SAIL_717_D10	LP SAIL	TTTTTGTTTTGGAGCTTGCTC
	RP SAIL	AAGATGTTGGCTCCAACAATG
LB3 for SAIL lines genotyping	LB SAIL	GCCTTTTCAGAAATGGATAAATAGCCTTGCTTCC
