# Supplementary material for: Arabidopsis tonoplast intrinsic protein and vacuolar H+-adenosinetriphosphatase reflect vacuole dynamics during development of syncytia induced by the beet cyst nematode Heterodera schachtii
Source: Protoplasma. 2018 Sep 5;256(2):419–29. doi: 10.1007/s00709-018-1303-4 (PMC6510842; doi:10.1007/s00709-018-1303-4)
Supplement: Supplementary file 3 — (PDF 94.4 kb) [file 709_2018_1303_MOESM3_ESM.pdf]

Supplementary Table S2 Real-time qPCR conditions

Temperature	Time
PCR	
95 °C	2 min
40 cycles:	
95 °C	15 s
60 °C	30 s
Melting curve	
60 °C -> 95 °C (in 0.5 °C inc.)	5 s / step
