# Supplementary material for: Arabidopsis tonoplast intrinsic protein and vacuolar H+-adenosinetriphosphatase reflect vacuole dynamics during development of syncytia induced by the beet cyst nematode Heterodera schachtii
Source: Protoplasma. 2018 Sep 5;256(2):419–29. doi: 10.1007/s00709-018-1303-4 (PMC6510842; doi:10.1007/s00709-018-1303-4)
Supplement: Supplementary file 4 — (PDF 64.8 kb) [file 709_2018_1303_MOESM4_ESM.pdf]

Supplementary Table S3 Bioinformatics meta-analysis of nematode induced changes in expression of major intrinsic proteins and V-ATPase subunits genes published in transcriptomic studies by Szakasits et al. (2009) and Matuszkiewicz et al. (2018). Statistically significant gene expression changes in transcriptome analyses ($p<0.05$) are marked green and red (down- and up-regulated, respectively). "No" indicates insignificant changes

**Major intrinsic proteins**	**Gene**	**TAIR**	**Szakasits et al. 2009**	**Matuszkiewicz et al. 2018**
			**log$_2$FC**	**log$_2$FC**
**T**onoplast **I**ntrinsic **P**rotein	*TIP1;1*	At2g36830	-3.60	-2.48
**T**onoplast **I**ntrinsic **P**rotein	*TIP1;2*	At3g26520	-8.50	-1.48
**T**onoplast **I**ntrinsic **P**rotein	*TIP1;3*	At4g01470	No	No
**T**onoplast **I**ntrinsic **P**rotein	*TIP2;1*	At3g16240	No	-0.90
**T**onoplast **I**ntrinsic **P**rotein	*TIP2;2*	At4g17340	-8.40	-3.03
**T**onoplast **I**ntrinsic **P**rotein	*TIP2;3*	At5g47450	-9.40	-2.54
**T**onoplast **I**ntrinsic **P**rotein	*TIP3;1*	At1g73190	No	No
**T**onoplast **I**ntrinsic **P**rotein	*TIP3;2*	At1g17810	No	1.33
**T**onoplast **I**ntrinsic **P**rotein	*TIP4;1*	At2g25810	-0.90	-2.56
**T**onoplast **I**ntrinsic **P**rotein	*TIP5;1*	At3g47440	No	No
**N**odulin26-like **I**ntrinsic **P**rotein	*NIP1:1*	At4g19030	-0.40	-1.58
**N**odulin26-like **I**ntrinsic **P**rotein	*NIP1:2*	At4g18910	No	1.31
**N**odulin26-like **I**ntrinsic **P**rotein	*NIP2:1*	At2g34390	No	-0.60
**N**odulin26-like **I**ntrinsic **P**rotein	*NIP3:1*	At1g31880	No	No
**N**odulin26-like **I**ntrinsic **P**rotein	*NIP4:1*	At5g37810	No	No
**N**odulin26-like **I**ntrinsic **P**rotein	*NIP4:2*	At5g37820	No	1.32
**N**odulin26-like **I**ntrinsic **P**rotein	*NIP5:1*	At4g10380	-2.20	-0.55
**N**odulin26-like **I**ntrinsic **P**rotein	*NIP6:1*	At1g80760	No	-1.10
**N**odulin26-like **I**ntrinsic **P**rotein	*NIP7:1*	At3g06100	No	No
**P**lasma membrane **I**ntrinsic **P**rotein	*PIP1:1*	At3g61430	-10.80	-2.02
**P**lasma membrane **I**ntrinsic **P**rotein	*PIP1:2*	At2g45960	-7.40	-1.56
**P**lasma membrane **I**ntrinsic **P**rotein	*PIP1:3*	At1g01620	-8.60	-0.97
**P**lasma membrane **I**ntrinsic **P**rotein	*PIP1:4*	At4g00430	-5.10	No
**P**lasma membrane **I**ntrinsic **P**rotein	*PIP1:5*	At4g23400	-9.00	-1.93
**P**lasma membrane **I**ntrinsic **P**rotein	*PIP2:1*	At3g53420	-9.20	-2.29
**P**lasma membrane **I**ntrinsic **P**rotein	*PIP2:2*	At2g37170	No	-2.63
**P**lasma membrane **I**ntrinsic **P**rotein	*PIP2:3*	At2g37180	No	-1.00
**P**lasma membrane **I**ntrinsic **P**rotein	*PIP2:4*	At5g60660	-6.70	-2.87
**P**lasma membrane **I**ntrinsic **P**rotein	*PIP2:5*	At3g54820	-1.20	2.14
**P**lasma membrane **I**ntrinsic **P**rotein	*PIP2:6*	At2g39010	-1.90	1.13
**P**lasma membrane **I**ntrinsic **P**rotein	*PIP2:7*	At4g35100	No	-0.64

**P**lasma membrane **I**ntrinsic **P**rotein	*PIP2:8*	At2g16850	No	-0.58
**S**mall basic **I**ntrinsic **P**rotein	*SIP1:1*	At3g04090	No	-0.47
**S**mall basic **I**ntrinsic **P**rotein	*SIP1:2*	At5g18290	1.30	-0.62
**S**mall basic **I**ntrinsic **P**rotein	*SIP2:1*	At3g56950	No	-0.80

**V-ATPase proton pump**	**Gene**	**TAIR**	**Szakasits et al. 2009**	**Matuszkiewicz et al. 2018**
			**log$_2$FC**	**log$_2$FC**
V-ATPase peripheral V1 complex	*AtVHA-A*	At1g78900	No	-0.44
V-ATPase peripheral V1 complex	*AtVHA-B1*	At1g76030	No	-0.45
V-ATPase peripheral V1 complex	*AtVHA-B2*	At4g38510	No	No
V-ATPase peripheral V1 complex	*AtVHA-B3*	At1g20260	No	-0.67
V-ATPase peripheral V1 complex	*AtVHA-C*	At1g12840	No	No
V-ATPase peripheral V1 complex	*AtVHA-D*	At3g58730	No	No
V-ATPase peripheral V1 complex	*AtVHA-E1*	At4g11150	No	-0.45
V-ATPase peripheral V1 complex	*AtVHA-E2*	At3g08560	No	No
V-ATPase peripheral V1 complex	*AtVHA-E3*	At1g64200	-1.20	-1.08
V-ATPase peripheral V1 complex	*AtVHA-F*	At4g02620	No	-0.59
V-ATPase peripheral V1 complex	*AtVHA-G1*	At3g01390	No	-0.51
V-ATPase peripheral V1 complex	*AtVHA-G2*	At4g23710	No	-1.04
V-ATPase peripheral V1 complex	*AtVHA-G3*	At4g25950	No	No
V-ATPase peripheral V1 complex	*AtVHA-H*	At3g42050	No	No
V-ATPase membrane-integral V0 complex	*AtVHA-a1*	At2g28520	No	No
V-ATPase membrane-integral V0 complex	*AtVHA-a2*	At2g21410	-0.90	No
V-ATPase membrane-integral V0 complex	*AtVHA-a3*	At4g39080	No	-0.61
V-ATPase membrane-integral V0 complex	*AtVHA-c1*	At4g34720	No	-0.91
V-ATPase membrane-integral V0 complex	*AtVHA-c2*	At1g19910	No	-0.81
V-ATPase membrane-integral V0 complex	*AtVHA-c3*	At4g38920	No	-0.44
V-ATPase membrane-integral V0 complex	*AtVHA-c4*	At1g75630	No	-0.49
V-ATPase membrane-integral V0 complex	*AtVHA-c5*	At2g16510	No	No
V-ATPase membrane-integral V0 complex	*AtVHA-c"1*	At4g32530	No	No
V-ATPase membrane-integral V0 complex	*AtVHA-c"2*	At2g25610	No	No
V-ATPase membrane-integral V0 complex	*AtVHA-d1*	At3g28710	No	No
V-ATPase membrane-integral V0 complex	*AtVHA-d2*	At3g28715	No	No
V-ATPase membrane-integral V0 complex	*AtVHA-e1*	At5g55290	0.60	-0.69
V-ATPase membrane-integral V0 complex	*AtVHA-e2*	At4g26710	No	-0.86
